# Comparison of Safety of RADial comPRESSion Devices: A Multi-Center Trial of Patent Hemostasis following Percutaneous Coronary Intervention from Conventional Radial Access (RAD-PRESS Trial)

**DOI:** 10.3390/diagnostics13010143

**Published:** 2023-01-01

**Authors:** Balazs T. Nemeth, Istvan Hizoh, Fanni Nowotta, Zoltan Ruzsa, Tibor Szuk, Peter Kulyassa, Gabor A. Fulop, Fanni E. Szablics, David Becker, Bela Merkely, Istvan F. Edes

**Affiliations:** 1 Heart and Vascular Center, Semmelweis University, 1122 Budapest, Hungary; 2 2nd Department of Internal Medicine and Cardiology Center, University of Szeged, 6720 Szeged, Hungary; 3 Department of Cardiology and Cardiac Surgery, University of Debrecen, 4032 Debrecen, Hungary

**Keywords:** radial compression, radial artery occlusion, patent hemostasis, transradial approach

## Abstract

Although radial access is the current gold standard for the implementation of percutaneous coronary interventions (PCI), post-procedural radial compression devices are seldom compared with each other in terms of safety or efficacy. Our group aimed to compare a cost effective and potentially green method to dedicated radial compression devices, with respect to access site complications combined in a device oriented complex endpoint (DOCE), freedom from which served as our primary endpoint. Patients undergoing PCI were randomized to receive either the cost effective or a dedicated device, either of which were removed using patent hemostasis. Twenty-four hours after the procedure, radial artery ultrasonography was performed to evaluate the access site. The primary endpoint was assessed using a non-inferiority framework with a non-inferiority margin of five percentage points, which was considered as the least clinically meaningful difference. The cost-effective technique and the dedicated devices were associated with a comparably low rate of complications (freedom from DOCE: 83.3% vs. 70.8%, absolute risk difference: 12.5%, one-sided 95% confidence interval (CI): 1.11%). Composition of the DOCE (i.e., no complication, hematoma, pseudoaneurysm, and radial artery occlusion) and compression time were also assessed in superiority tests as secondary endpoints. Both the cost-effective technique and the dedicated devices were associated with comparably low rates of complications: *p* = 0.1289. All radial compression devices performed similarly when considering the time to complete removal of the respective device (120.0 (inter-quartile range: 100.0–142.5) for the vial vs. 120.0 (inter-quartile range: 110.0–180) for the dedicated device arm, with a median difference of [95% CI]: 7.0 [−23.11 to 2.00] min, *p* = 0.2816). In conclusion, our cost-effective method was found to be non-inferior to the dedicated devices with respect to safety, therefore it is a safe alternative to dedicated radial compression devices, as well as seeming to be similarly effective.

## 1. Introduction

Radial artery access is recommended as the standard approach for invasive coronary angiography and percutaneous coronary interventions (PCI) in the European Society of Cardiology Guidelines due to its effectiveness and safety compared with femoral access [1]. Complications such as occlusion or (pseudo)aneurysm of the radial artery and hematoma formation at the access site, however, are prevalent complications following transradial procedures. Radial artery occlusion (RAO) was initially found to be occurring in up to 33% of transradial procedures [2], which later decreased substantially according to more recent results [3]. The development of patent hemostasis [4,5] might have had an impact on these favorable changes. RAO remains, however, the most frequent vascular complication following radial access. As many of the suspected predictors of RAO are difficult to be addressed [6,7,8,9], modifiable procedural and post-procedural risk factors such as inadequate anticoagulation and occlusive or prolonged hemostasis should be reduced to further mitigate its incidence [10,11].

A multitude of compression devices have been developed, the two most frequently applied solutions of which are mechanical and pneumatic. The performance of these dedicated devices are seldom compared with each other in terms of safety or efficacy, therefore there is limited evidence to support the use of any single hemostatic method to prevent RAO and other access site complications after percutaneous coronary procedures [12,13].

**Figure 1:**
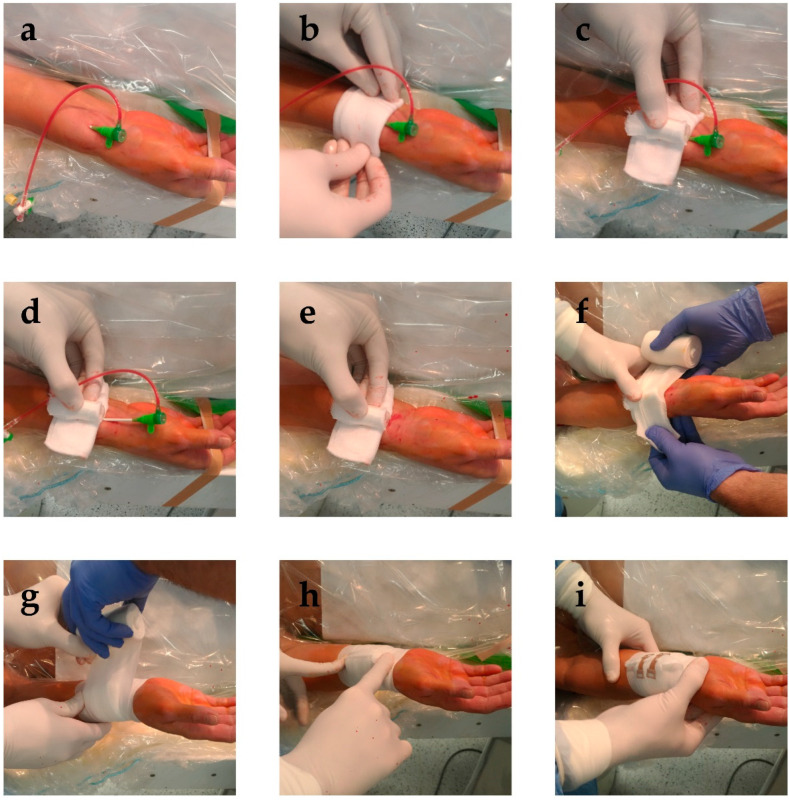
Placement of the vial compression. (**a**) After completion of the procedure, the surroundings of the access site are cleaned. (**b**) A sterile gauze is placed above the access site, with the sheath still in place. (**c**) The reused vial wrapped in sterile gauze is placed above the access site, with its longer axis parallel to the radial artery. (**d**) The sheath is then removed, (**e**) while applying sufficient pressure to prevent bleeding. (**f**,**g**) An elastic bandage is then wound six times around the vial, (**h**) after which it is cut above the access site. (**i**) After the loose end of the elastic bandage is secured, the compression is functional.

Our institutions historically utilized a cost effective and potentially green method to compress the radial artery following invasive angiography and PCI: we reuse the glass vial of the intravenous medication heparin, cover it in a sterile gauze and place it above the access site, compress it with an elastic bandage, and then secure it with two clamps (Figure 1). We designed the RAD-PRESS trial to assess the safety of this vial technique compared with two major dedicated industrial solutions.

## 2. Materials and Methods

The RAD-PRESS trial was designed as a multicenter, prospective, randomized non-inferiority safety trial aiming to assess safety of a cost-effective radial compression device compared with dedicated and widely used competitors in patients undergoing PCI from radial access. Inclusion and exclusion criteria for the study are listed in Table 1. The primary endpoint for the study was freedom from a device oriented complex endpoint (DOCE) comprising radial artery occlusion, formation of a radial artery aneurysm, and bleeding from or hematoma of the access site, all within 24 h after implementing the below detailed patent hemostasis protocol following the index procedure. Secondary endpoints were components of the primary endpoint. Compression time needed to achieve primary hemostasis was also recorded for every subject. All procedures related to the trial were performed after informed consent was obtained from participants. The RAD-PRESS trial was catalogued and authorized by the Hungarian National Institute of Pharmacy and Nutrition under number: OGYÉI/24194/2018.

**Figure 2:**
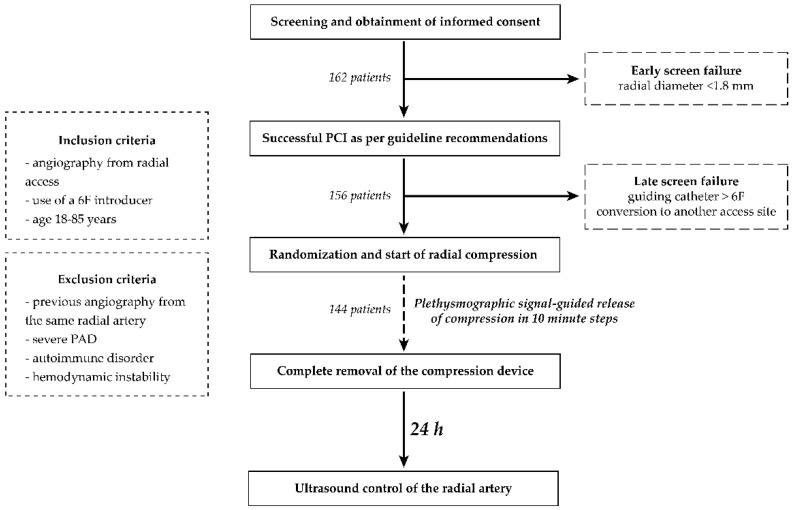
Summary of the study protocol.

All elective patients between age 18 and 85, referred to the cardiac catheterization laboratories involved in the trial, were invited to participate in the study unless fulfilling an exclusion criterion (Figure 2). After consent was obtained, radial artery diameter was assessed using vascular ultrasound. Patients with radial artery diameter smaller than 1.8 mm were excluded from the trial (early screen failure) [7]. Diagnostic coronary angiography was then completed using a 6F hydrophilic introducer sheath and 5F diagnostic catheters as required by the anatomical situation, and patients not requiring PCI were also excluded (late screen failure). Late screen failure occurred if an alternative access site or a guiding catheter larger than 6F was required for any reason during the intervention as well (Figure 2). Baseline demographic, clinical and procedural data of the patients were recorded.

Simple randomization to a screw-knob/pneumatic balloon (together: Dedicated) Device or the Vial technique was done after completion of the intervention in a 1:1 ratio between the groups as well as within Dedicated Devices. Compression devices were applied as follows or as detailed in Figure 1 for the Vial group. Compression rate of the screw-knob device used in this trial can be set in 9 steps. By default, we used step 6 for all patients. If bleeding was visible from the puncture site at this setting, compression was increased by 2 steps. For the pneumatic balloon device, the default air volume used for compression was 12 mL. Similar to the screw-knob device, on occasion of visible bleeding from the access site, a further 2 mL of air was added. The increase in compression described above sufficed for the patients in whom the default settings were insufficient with these devices.

All compressive devices were attempted to be removed utilizing a patent hemostasis protocol based on the one used in PROPHET and PROPHET II trials [4,14] as follows (Figure 2). Pulse oximetry was used to guide the release of compression in every 10 min by releasing one step, deflating 2 mL of air, or unwinding two rotations of the wrapping for the screw-knob, pneumatic balloon, and the Vial technique, respectively. The ipsilateral ulnar artery was compressed manually during the first attempt at decompression until plethysmographic signal returned on the pulse oximeter. On occasion of bleeding from the access site as a result of this maneuver, compression was increased again by increasing one step, inflation of 2 mL of air, or rewinding two rotations of the wrapping for the screw-knob, pneumatic balloon, and the Vial technique, respectively. Decompression was reattempted 10 min later. For further decompressions, the above steps were repeated. Time required to completely remove the compression device was set at 120 min, and was recorded for every subject. Radial artery ultrasound was repeated by an investigator blinded to the study groups 24 h after the index procedure to evaluate the access site with respect to the DOCE. Hematoma formation was assessed as well at 24 h based on the EASY hematoma scale [15].

From a statistical aspect, the study was primarily designed using a non-inferiority framework. The null hypothesis was that the rate of the primary endpoint (DOCE) for the heparin Vial group is 5.0 percentage points lower than that in the Dedicated Device group, i.e., the non-inferiority margin was set at 5–percentage points which was considered as the least clinically meaningful difference. The treatment arms were compared using the 1-sided 95% confidence interval (CI) approach.

Though our study was conceived as a non-inferiority analysis, the data were also evaluated for possible superiority. Therefore, the treatment arms were compared using the 2-sided 95% CI approach and a 2 tailed *p* value less than 0.05 was considered statistically significant. The primary endpoint was analyzed using Fisher’s exact test. As to the secondary endpoints, composition of the DOCE (i.e., no complication, hematoma, pseudoaneurysm, and radial artery occlusion) was assessed by the exact Cochran–Armitage test in order to detect trends in data from 2 × 4 contingency tables, whereas compression times were compared using the exact Mann–Whitney test. All statistical analyses and graphical interpretations of the results were carried out with R version 4.2.1 (R Foundation for Statistical Computing, Vienna, Austria) using the coin 1.4 2, dplyr 1.0.9, epiR 2.0.50, fBasics 3042.89.2, ggplot2 3.3.6, and Hmisc 4.7-0 additional packages. All analyses were done on an intention-to-treat basis.

## 3. Results

### 3.1. Clinical Data

The baseline characteristics of the patients are presented in Table 1. There were no significant differences among the groups.

### 3.2. Efficacy and Safety Data

The primary endpoint was met in the Dedicated Device and the Vial groups similarly (Table 2). Distribution of the components of the DOCE were also comparable between the groups with hematoma formation of EASY Grade 1 and 2 being the most prevalent complication (Table 2).

**Figure 3:**
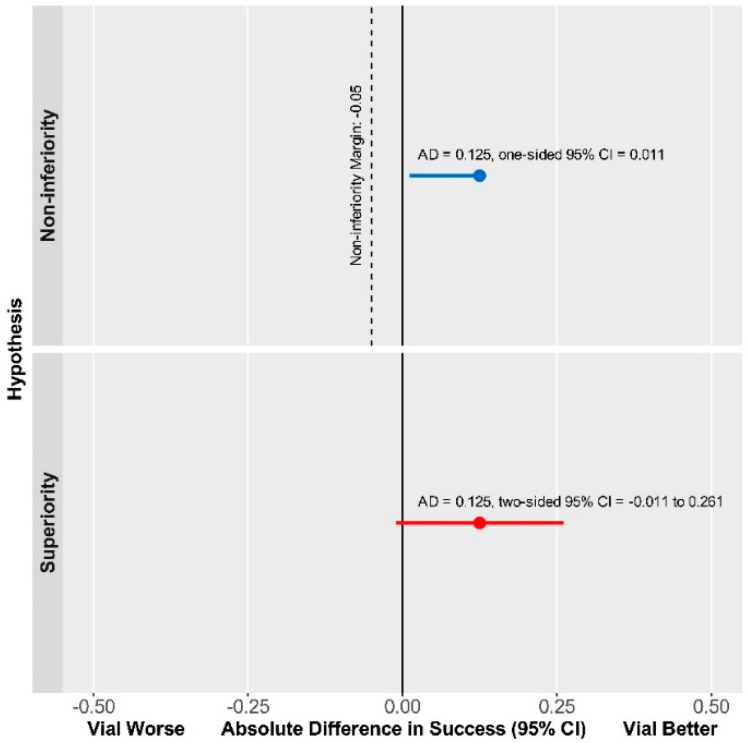
The Vial technique is non-inferior compared with Dedicated Device with respect to DOCE.

The median difference in compression time was -7.0 min (median difference [95% CI]: 7.0 [-23.11 to 2.00] min., *p* = 0.2816). Therefore, the Vial technique was found to be non-inferior to Dedicated Devices with respect to the DOCE, while superiority was not reached (Figure 3).

## 4. Discussion

In the present study we provide evidence that a cost-effective, and through the recycling glass vials, a potentially green alternative is non-inferior to dedicated radial compression devices in terms of the avoidance of complications of the access site when performing patent hemostasis in patients undergoing percutaneous coronary interventions from a conventional radial access.

A multitude of radial compression devices have been developed by companies around the globe. Safety and efficacy of some of these have been investigated in comparative studies [16,17,18,19], but all of the investigated devices were dedicated to the radial access site. This is the first instance in which a cost-effective heparin vial method, which also satisfies the increasingly more important aspect of environmental awareness, is compared with a dedicated radial compression device.

The primary endpoint was met in a similar number of patients in both the Vial and Dedicated Device groups (Table 2). Furthermore, secondary endpoints, i.e., hematoma formation, pseudoaneurysm, or RAO rates (Table 2) also did not differ among the groups. Our data are in line with previously found complication rates of 5–18% for hematoma [16,17,18,19]. RAO, on the other hand, at a rate of 1% was considerably less prevalent in our cohort than in previous comparative studies, which reported RAO to occur in 2–14% [16,17,18,19]. This result might be attributable to the patent hemostasis protocol utilized by our group during the investigation. Although distal radial access has emerged in recent years as a potentially effective method to reduce access site complication rates including RAO, according to the latest data coming from a large, randomized trial, it does not provide benefit in this regard compared with conventional radial access when hemostasis is adequately managed [20]. This result is in line with our data in the RAD-PRESS trial, in which we utilized a rigorous patent hemostasis protocol and produced an RAO rate of 1.4%. Overall, our data suggests that both approaches utilized in the study achieved safe hemostasis following PCI, the Vial technique being non-inferior to Dedicated Device technique in this regard (Figure 3).

To our knowledge, there are no published data about the treatment effect of heparin vials as compression devices. However, based on our single-center, unpublished registry data, we assumed a primary endpoint frequency of 70.0% and 83.0% in the Dedicated Device and Vial groups, respectively. Thus, the enrollment of 134 patients (67 in each arm) would have provided the study with a statistical power of 80.0% to detect a 5.0% decrease in the rate of the primary endpoint at a 1-sided α level of 0.05. With the observed event rates and final sample size of 144 patients (72 in each group), the study has an a posteriori statistical power of 80.1% to detect the null hypothesis at a 1-sided α level of 0.05.

In conclusion, our data suggest that the cost-effective and green vial technique is similarly safe and potentially similarly effective as dedicated devices in compressing the radial artery following percutaneous coronary interventions from conventional radial access. The environmentally friendly recycling of heparin vials therefore is a viable option for the compression of the radial artery.

## 5. Limitations

There are a few limitations of the trial discussed herein that need to be addressed. First, a follow-up of only 24 h is not enough to detect all RAOs, as some of these occur later. A longer follow-up of at least 30 days would potentially result in a higher rate of RAO. A further limiting factor is that the Vial technique cannot be undoubtedly standardized, therefore user variability might have had a role in the results of the Vial group. Moreover, as superficial and not just deep hematomas were counted, these might have been over diagnosed in this trial, hence the relatively large number of hematomas in comparison with other, large, randomized trials. The observed rate of other complications combined in the DOCE at 24 h was very low, which mandates caution when interpreting our results despite the study being adequately powered statistically. Larger trials with more power are warranted to fully uncover the rate of complications when using the Vial technique.

## Figures and Tables

**Table 1:** Baseline Demographic, Clinical, and Procedural Characteristics.

Variable	Dedicated Device (*n* = 72)	Vial (*n* = 72)
Age Median (IQR) (years)	66.5 (58.0–74.3)	65.5 (57.8–70.3)
Female	31 (43.1%)	24 (33.3%)
Hypertension	62 (86.1%)	65 (90.3%)
Diabetes mellitus	23 (31.9%)	31 (43.1%)
Hyperlipoproteinemia	59 (81.9%)	52 (72.2%)
Peripheral arterial disease	7 (9.7%)	4 (5.6%)
Impaired renal function ^1^	15 (20.8%)	11 (15.3%)
Acute coronary syndrome	22 (30.6%)	13 (18.1%)
Right radial artery	60 (83.3%)	63 (87.5%)
Radial artery diameter Median (IQR) (mm)	2.5 (2.1–2.8)	2.55 (2.1–3.0)

IQR—inter-quartile range; ^1^—GFR <60 mL/min.

**Table 2:** Results.

Outcome Measure	Dedicated Device (*n* = 72)	Vial (*n* = 72)	*p* Value (Two-Sided)
Primary endpoint			
Freedom from DOCE	51 (70.8%)	60 (83.3%)	0.11
Secondary endpoints	68 (94.4%)	69 (95.8%)	1.0
Distribution of outcomes			
No complication	51 (70.8%)	60 (83.3%)	
Hematoma	20 (27.8%)	11 (15.3%)	0.13
Pseudoaneurysm	1 (1.4%)	0 (0.0%)	
Radial artery occlusion	0 (0.0%)	1 (1.4%)	
Compression time (min)	120.0 (110.0–180)	120.0 (100.0–142.5)	0.28

DOCE—device oriented complex endpoint.

## Data Availability

The data are not publicly available due to constraints from the authority issuing the study authorization.
